# Factors associated with online media attention to research: a cohort study of articles evaluating cancer treatments

**DOI:** 10.1186/s41073-017-0033-z

**Published:** 2017-07-01

**Authors:** Romana Haneef, Philippe Ravaud, Gabriel Baron, Lina Ghosn, Isabelle Boutron

**Affiliations:** 1 INSERM, UMR 1153 Epidemiology and Biostatistics Sorbonne Paris Cité Center (CRESS), METHODS team, University of Paris Descartes, Centre d’Épidémiologie Clinique, AP-HP (Assistance Publique des Hôpitaux de Paris), Hôpital Hôtel Dieu, Paris, France; 2 Paris Descartes University, Sorbonne Paris Cité, Faculté de Médecine, Paris, France; 3 Centre d’Épidémiologie Clinique, AP-HP (Assistance Publique des Hôpitaux de Paris), Hôpital Hôtel Dieu, Paris, France; 4 French Cochrane Center, Paris, France; 5 Department of Epidemiology, Columbia University Mailman School of Public Health, New York, NY USA

**Keywords:** Cancer treatment, Media attention, Altmetric score, Journal impact factor, Press release, Open access

## Abstract

**Background:**

New metrics have been developed to assess the impact of research and provide an indication of online media attention and data dissemination. We aimed to describe online media attention of articles evaluating cancer treatments and identify the factors associated with high online media attention.

**Methods:**

We systematically searched MEDLINE via PubMed on March 1, 2015 for articles published during the first 6 months of 2014 in oncology and medical journals with a diverse range of impact factors, from 3.9 to 54.4, and selected a sample of articles evaluating a cancer treatment regardless of study design. Altmetric Explorer was used to identify online media attention of selected articles. The primary outcome was media attention an article received online as measured by Altmetric score (i.e., number of mentions in online news outlets, science blogs and social media). Regression analysis was performed to investigate the factors associated with high media attention, and regression coefficients represent the logarithm of ratio of mean (RoM) values of Altmetric score per unit change in the covariate.

**Results:**

Among 792 articles, 218 (27.5%) received no online media attention (Altmetric score = 0). The median [Q1–Q3] Altmetric score was 2.0 [0.0–8.0], range 0.0–428.0. On multivariate analysis, factors associated with high Altmetric score were presence of a press release (RoM = 10.14, 95%CI [4.91–20.96]), open access to the article (RoM = 1.48, 95%CI [1.02–2.16]), and journal impact factor (RoM = 1.10, 95%CI [1.07–1.12]. As compared with observational studies, systematic reviews were not associated with high Altmetric score (RoM = 1.46, 95%CI [0.74–2.86]; *P* = 0.27), nor were RCTs (RoM = 0.65, 95%CI [0.41–1.02]; *P* = 0.059) and phase I/II non-RCTs (RoM = 0.58, 95%CI [0.33–1.05]; *P* = 0.07). The articles with abstract conclusions favouring study treatments were not associated with high Altmetric score (RoM = 0.97, 95%CI [0.60–1.58]; *P* = 0.91).

**Conclusions:**

Most important factors associated with high online media attention were the presence of a press release and the journal impact factor. There was no evidence that study design with high level of evidence and type of abstract conclusion were associated with high online media attention.

**Electronic supplementary material:**

The online version of this article (doi:10.1186/s41073-017-0033-z) contains supplementary material, which is available to authorized users.

## Background

Global oncology spending reached $100 billion in 2014 [1], and more than 100,000 research articles are published every year in the field of cancer. It is important to evaluate the impact of this research. The most widely used indicator to measure the impact of research is the number of citations received for each published article [2, 3]. However, citations only measure the impact in the scientific community [4] but not on other important stakeholders such as policy makers, patients, and the general public [2]. Furthermore, this impact can be assessed only after a wait of months [5, 6].

New metrics have been developed to assess the impact of research and provide an indication of online media attention, data dissemination and effect of research across global community. For example, Altmetric was developed to measure the media attention an article receives online [7]. These metrics track online attention for a specific research through an output (e.g., journal article), an identifier linked to the output (e.g., digital object identifier (DOI)) and mentions in a source (e.g., online news outlets). Each article receives an Altmetric score measuring the number of mentions the article has received in online news outlets, science blogs and social media (Twitter, Facebook, Google+, etc.) to provide an indicator of the amount of online media attention [8]. The score is derived from an automated algorithm and represents a weighted count of the amount of attention received for a research output [9]. However, the Altmetric score is not the only factor of scholarly impact. This score is widely used by journal editors and researchers to analyze the effect of the research they publish within days after their publication [2, 10–13].

To our knowledge, no study has evaluated online media attention in the field of cancer. Therefore, we aimed to describe and identify the factors associated with online media attention of articles evaluating cancer treatments. Particularly, we aimed to determine whether more attention was received by studies with a high level of evidence [14–17]. We focused on studies evaluating treatments because they interest the scientific community and are important to healthcare professionals, policy makers, patients and caregivers.

## Methods

### Study design

We conducted a cohort study of articles reporting studies evaluating treatments in the field of cancer and published in high-impact-factor journals.

### Identification of articles

#### Search strategy

We screened the highest impact factor journals in the following categories: 50 in “Oncology”, 25 in “Medicine, General and Internal” and 25 in “Medicine, Research and Experimental” (Journal citation report 2013, Thomson Reuters). We selected the journals that were publishing clinical studies or systematic reviews of clinical studies or observational studies evaluating the effect of interventions on humans and identified 24 journals from “Oncology”, 17 from “Medicine, General and Internal” and 6 from “Medicine, Research and Experimental”. We then searched MEDLINE via PubMed on March 1, 2015 for articles published from January 1, 2014 to June 30, 2014 in the selected journals by using the following search strategy: “name of the journal” in the journal search field; “cancer” in title and abstract field; article type “randomized controlled trials”, “clinical trials”, “observational studies”, “meta-analysis” or “systematic reviews” and text availability “abstract”.

#### Eligibility criteria

We included all studies evaluating an intervention to improve the health of patients with any type of cancer, regardless of study design. These interventions could concern chemotherapy, targeted therapy, radiotherapy, surgery, hormone therapy, immunotherapy and supportive care (e.g., analgesics, antibiotics, antiretroviral, dietary supplements, multivitamins, vaccination). We excluded studies of diagnostics, screening, prognostic factors, biomarkers, correlation and gene, molecular and protein expression that did not evaluate any treatment. We also excluded animal studies and narrative reviews.

### Data extraction

An online data extraction form was developed and preliminarily tested on a sample of 30 articles. The following data were collected: journal type (i.e., cancer or general medical), study design (systematic reviews/meta-analyses (SRs/MAs), randomized controlled trials (RCTs), phase I/II non-randomized trials and observational studies), sample size and funding source (i.e., for profit, non-profit, both and not reported). The types of cancer and type of cancer treatments were classified according to the US National Cancer Institute” [18].

We determined whether the abstract conclusion favoured the study treatment, did not favour the study treatment or was neutral [19]. We checked whether there was an open access to the article on PubMed and recorded the online publication date on PubMed. Finally, we also checked whether the published article had issued a press release or not. For this purpose, we searched EurekAlert (online free database for science press releases: http://www.eurekalert.org/) using keywords from PubMed, online or journal publication date, journal name, authors’ first and last names and title.

Two researchers (RH, LG) with expertise in clinical epidemiology independently screened the titles and abstracts for 25% of the citations retrieved and extracted specific information. The reproducibility was very good (kappa > 0.9 for all items) (Additional file 1). Then, the remaining citations were divided among the two researchers for further screening and data extraction. The full text was retrieved to record the funding source when not reported in the abstract.

### Online media attention measured by Altmetric score

The primary outcome was the online media attention measured by the Altmetric score. The Altmetric Web-based application tracks the attention scholarly articles receive online by using data from three main categories of sources: social media (i.e., Twitter, Facebook, Google+, Pinterest and blogs); traditional media (i.e., mainstream, such as *The Guardian*, *New York Times*, and science-specific, such as *New Scientist* and *Scientific American*) and online reference managers (i.e., Mendeley and CiteULike) [20]. This score, providing a quantitative measure of attention a scholarly article receives online, is derived from an automated algorithm. The score is weighted by the relative coverage of each published research article in each type of source (e.g., news, Twitter) [9]. For example, an average newspaper story is more likely to bring attention to the research article than an average tweet [9]. Additional file 2 provides details on how the Altmetric score is calculated.

The effect of time is important in exposure of media attention to the article [11]. In general, the published article receives maximum online attention within 6 months of its publication. Each mention of an article on online sources affects the Altmetric score. Therefore, we chose a delay of at least 10 months from the last publication date (June 30, 2014) to the Altmetric search date (May 1, 2015) to allow for sufficient exposure for a stable Altmetric score.

We searched Altmetric Explorer [7] by using the PubMed unique identifier (PMID) for the selected articles (Altmetric search date: May 1, 2015). Then, we downloaded the Altmetric score and number of news items, science blogs, tweets, Facebook posts, Google+ posts, Mendeley readers, CiteULike and some other sources where the published article was mentioned.

### Statistical analysis

Qualitative variables are described with frequencies and percentages (%). Quantitative variables are described with medians [Q1–Q3]. We used the negative binomial GEE model to study the association of explanatory variables and Altmetric score. Regression coefficients represent the logarithm of the ratio of mean (RoM) values of the Altmetric score per unit change in the covariate. We chose this model to explain the wide dispersion of Altmetric score (greater variance than the mean). Using a function “offset”, we adjusted for the duration between online publication dates of articles (or journal publication date if the online publication date was greater than journal publication date) and the search date for Altmetric score (May 1, 2015) to account for the same post-publication exposure period. Clustering due to journals was accounted for by adding an exchangeable correlation structure to the model.

Univariate and multivariate analyses involved the following pre-specified explanatory variables: (1) journal impact factor, (2) study design in four classes (i.e., SR/MA, RCT, phase I/II non-randomized trial and observational study[as a referent group]), (3) abstract conclusion (in favour of study treatment (yes vs no [not in favour of study treatment and neutral]), (4) funding source (for profit [profit, both (profit and non-profit)] vs non-profit [non-profit, none and not reported]), (5) open access to the article (yes vs no) and (6) presence of a press release (yes vs no). All these variables were entered in the multivariate model to assess the association of each variable with high Altmetric score (controlling for the other variables in the model). Results are expressed as RoMs with 95% confidence intervals (95%CIs) for both univariate and multivariate analysis. Statistical analysis involved use of SAS for Windows 9.4 (SAS Inst., Cary, NC).

## Results

### General characteristics of selected articles

**Fig. 1:**
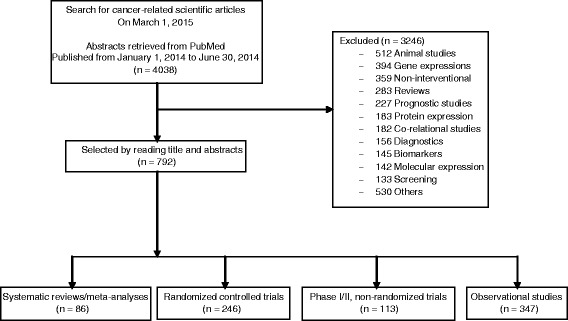
Flow diagram of articles evaluating cancer treatments

Among 47 selected journals, 4038 citations were retrieved. The 792 articles identified were published in 31 journals with a diverse range of impact factors, from 3.9 to 54.4 (Fig. 1). At least one article was selected among the 31 journals; the median [Q1–Q3] of included articles per journal was 10.0 [3.0–42.0]. Selected journals with the included number of articles are detailed in Additional file 3. The general characteristics of the articles selected are in Table 1. The median [Q1–Q3] of the journal impact factor of selected articles was 5.3[4.8–16.4]. Overall, 347 articles (44%) described observational studies, 246 (31%) RCTs, 113 (14%) phase I/II, non-randomized trials and 86 (11%) SRs/MAs. Most were published in cancer journals (*n* = 739, 93%). Among the 792 articles, in 523 (66%), the abstract conclusion was in favour of the study treatment, the funding source was for profit for 268 (34%), and 462 (58%) had open access to the article. Overall, only 56 (7%) of the articles had a press release.

**Table 1:** General characteristics of articles

Category	Total (*n* = 792)
Type of journal, *n* (%)	
− Cancer	739 (93.3)
− General medical	53 (6.7)
Journal impact factor, median [Q1–Q3]	5.3 [4.8–16.4]
Study design	
− Systematic review/meta-analysis	86 (10.9)
− Randomized controlled trial	246 (31.1)
− Phase I/II, non-randomized trial	113 (14.3)
− Observational study	347 (43.8)
Cancer type by organ, *n* (%)	
− Digestive system	168 (21.2)
− Breast	135 (17.0)
− Lungs	82 (10.4)
− Blood	71 (8.9)
− Prostate	53 (6.7)
− Female reproductive organ	44 (5.6)
− Others	239 (30.2)
Type of cancer treatment, *n* (%)	
− Chemotherapy	212 (26.7)
− Targeted therapy	88 (11.1)
− Radiotherapy	69 (8.7)
− Surgery	44 (5.5)
− Hormone therapy	28 (3.5)
− Immunotherapy	4 (0.5)
− Supportive care	197 (25.0)
− Others	150 (19.0)
Sample size, median [Q1–Q3]^a^	181.0 [48.5–1010.5]
Type of abstract conclusion	
− In favour of study treatment	523 (66.0)
− Not in favour of study treatment	269 (34.0)
Funding source, *n* (%)	
− Non-profit	418 (52.8)
− Profit^b^	268 (33.8)
− Not reported	106 (13.4)
Altmetric score, median [Q1–Q3]	2.0 [0.0–8.0]
Open access	
− Yes	462(58)
− No	330(42)
Press-release	
− Yes	56(7)
− No	736(93)

^a^Excluding the sample size of systematic reviews/meta-analyses

^b^12.2% is partially profit and non-profit

### Description of online media attention measured by Altmetric score

**Fig. 2:**
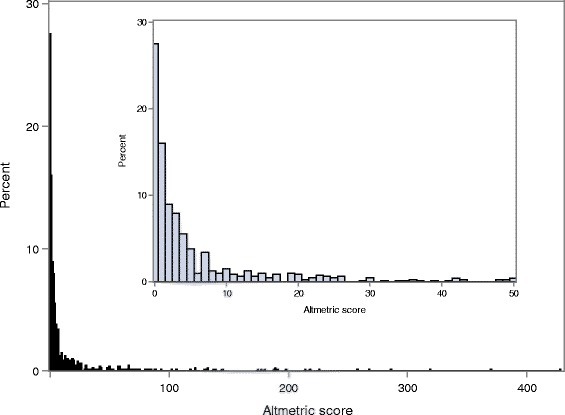
Distribution of Altmetric score for articles (*n* = 792) [Inset graph limited to articles with an Altmetric score ≤50]

The median [Q1–Q3] Altmetric score was 2.0 [0.0–8.0], range 0.0–428.0; 218 articles (27.5%) received no media attention (Altmetric score = 0). Figure 2 describes the overall distribution of Altmetric score of 792 articles.

Among 792 articles, 512 (64.7%) received a score between 1 and 50, 32 (4.0%) a score between 51 and 100, 21 (2.7%) a score between 101 and 200 and only 9 (1.1%) a score >200.

**Fig. 3:**
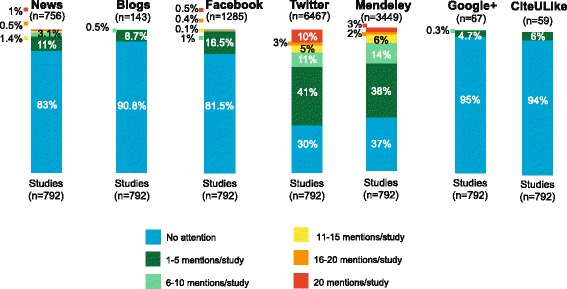
Online media attention of articles by sources (*n* = 792)

Figure 3 describes the amount of attention that studies received in different online media sources. Overall, there were 756 news outlets, 143 science blogs, 1285 facebook posts, 6467 tweets and 3449 Mendeley readers. In this figure, each bar represents the proportion of studies with no mention or attention (sky blue), 1–5 mentions per study (dark green), 6–10 mentions per study (jade green), 11–15 mentions per study (yellow), 16–20 mentions per study (orange) and 20 mentions per study (red). For example, in news media, 83% studies (657/792) received no attention, 11% (87/792) were mentioned 1–5 times, 3.1% (25/792) were mentioned 6–10 times, 1.4% (11/792) were mentioned 11–15 times, 0.5% (4/792) were mentioned 16–20 times, and only 1% (8/792) were mentioned 20 times.

### Factors associated with online media attention

On multivariate analysis, the factors associated with a high Altmetric score were presence of a press release (RoM = 10.14, 95%CI [4.91–20.96]; *P*˂0.0001), i.e., articles with press-release seemed to have 10.1 times increase in mean Altmetric score), open access to the article (RoM = 1.48, 95%CI [1.02–2.16]; *P =* 0.041), non-profit funding (RoM = 1.45, 95%CI [1.08–1.94]; *P =* 0.012) and journal impact factor (RoM = 1.10, 95% [1.07–1.12]; *P*˂0.0001), i.e., 1-point increase in impact factor has a 10% increase in mean Altmetric score (for instance a journal with an impact factor equal to 2), and a journal with an impact factor equal to 12 with a difference of 10 point in impact factor have an expected Altmetric score multiplied by 2.5 (150% increase for 10 points) (Table 2).

**Table 2:** Factors associated with online media attention (i.e., Altmetric score) of articles (*n* = 792)

		Univariate analysis			Multivariate analysis		
		RoM	95%CI	*P* value	RoM	95%CI	*P* value
Journal impact factor	(One unit)	1.11	[1.07–1.14]	<0.0001	1.10	[1.07–1.12]	<0.0001
Study design	• RCT vs observational study	1.02	[0.78–1.32]	0.9259	0.65	[0.41–1.02]	0.0593
	• Phase I/II, non-randomized trial vs observational study	0.46	[0.34–0.62]	<0.0001	0.58	[0.33–1.05]	0.0715
	• SR/MA vs observational study	0.97	[0.70–1.34]	0.8381	1.46	[0.74–2.86]	0.2724
Abstract conclusion	In favour of study treatment (yes vs no)	1.34	[1.04–1.74]	0.0254	0.97	[0.60–1.58]	0.9134
Funding source	Non-profit vs for profit	1.30	[0.97–1.73]	0.0773	1.45	[1.08–1.94]	0.0126
Open access	Yes vs no	1.72	[1.27–2.33]	0.0005	1.48	[1.02–2.16]	0.0418
Press release	Yes vs no	11.61	[6.78–19.87]	<0.0001	10.14	[4.91–20.96]	<0.0001

*RoM* ratio of mean

Systematic reviews (SR/MA) were not associated with high Altmetric score (RoM = 1.46, 95%CI [0.74–2.86]; *P* = 0.27) as compared with observational studies, nor were RCTs (RoM = 0.65, 95%CI [0.41–1.02]; *P* = 0.059) and phase I/II, non-RCTs (RoM = 0.58, 95%CI [0.33–1.05]; *P* = 0.07) as compared with observational studies. The articles with abstract conclusions favouring study treatments were not associated with high Altmetric score (RoM = 0.97, 95%CI [0.60–1.58]; *P* = 0.91).

Further details of means and medians for each explanatory variable associated with Altmetric score are in Table 3.

**Table 3:** Mean, median and [min–max] for explanatory variables associated with Altmetric score (*n* = 792)

Explanatory variables	Sub-categories	Mean (SD)	Median [Q1–Q3]	[Min–max]
Study design	SR/MA	14.9 (37.0)	3.5 [1.0–10.0]	[0.0–268.0]
	RCT	20.7 (50.5)	3.0 [0.0–16.0]	[0.0–428.0]
	Phase I/II, non-RCT	6.5 (17.2)	2.0 [0.0–4.0]	[0.0–139.0]
	Observational study	13.4 (39.7)	2.0 [0.0–7.0]	[0.0–319.0]
Abstract conclusion	In favour of study treatment	16.6 (44.8)	2.0 [0.0–9.0]	[0.0–428.0]
	Not in favour of study treatment	11.5 (32.5)	2.0 [0.0–7.0]	[0.0–319.0]
Funding source	Profit	13.9 (41.1)	2.0 [0.0–9.0]	[0.0–370.0]
	Non-profit	15.4 (41.1)	2.0 [0.0–8.0]	[0.0–428.0]
Open access	Yes	17.9 (49.3)	3.0 [1.0–8.0]	[0.0–428.0]
	No	10.6 (24.8)	1.5 [0.0–8.0]	[0.0–258.0]
Press release	Yes	118.6 (87.5)	84.5 [58.0–144.5]	[29.0–428.0]
	No	7.0 (19.0)	2.0 [0.0–5.0]	[0.0–268.0]

*SR/MA* systematic review/meta-analysis, *RCT* randomized controlled trial

## Discussion

This study describes the online media attention of 792 articles evaluating cancer treatments and identified associated factors. Almost one third of these studies received no media attention in terms of Altmetric score. The presence of a press release, open access to the article, non-profit funding source and journal impact factor were associated with high online media attention. There was no evidence that study design with a high level of evidence and type of abstract conclusion were associated with high online media attention.

To our knowledge, this is the first study describing the online media attention to articles evaluating cancer treatments and systematically determining the associated factors. Previous studies have mainly focussed on citation analysis to determine research impact within a speciality such as oncology [21], gastric cancer [22], general surgery [23], obstetrics and gynaecology [24] and urology [25].

Our results are consistent with previous studies showing that press releases are associated with the subsequent publication of newspaper stories [26, 27] and open access to the article increases the citation counts [28]. For example, Altmetric issued a list of 100 articles published in 2015 which received the highest media attention; 42% had open access [29]. Research articles exploring the impact of the study design and quality on citations are conflicting. Patsopoulos et al showed that articles with a study design with a high level of evidence received relatively more citations than other study designs [3]. In contrast, other work found no convincing evidence that journals with higher citation publish trials of higher methodological quality [30].

### Implications

Our study has some important implications. First, it shows that online media attention does not warrant the high quality of research. In fact, news, blogs and social media may highlight research on the basis of perceptions of their potential appeal to patients and the public, not because of their rigorous methodology. Indeed, previous studies showed that the media is more likely to cover observational studies and less likely to report RCTs [31]. A high level of evidence may interest the scientific and medical community more than the public.

Second, factors related to the publication process such as the presence of press release, open access are strongly associated with online media attention and the subsequent publication of newspaper stories [26, 27]. This is important information for researchers when planning the dissemination of their results. To enhance the impact of their research, they should favour open access and disseminate press releases.

Third, there is some evidence showing that high online media attention is highly correlated with access to the scientific article and the number of scholarly citations the scientific article will receive [2]. Some studies from the fields of clinical pain [10], urology [32], neurointerventional surgery [33] and cardiovascular [34] and emergency medicine [35] have shown that disseminating research on social media will increase their access or views to their readers. Highly cited articles can be predicted by tweets occurring within the first 3 days of article publication [2]. Open access to the article increases the citation counts [28].

Finally, high online media attention to articles evaluating treatments can have an impact on public health. Previous studies have shown that dissemination of medical research in the mass media can affect patients, public, researchers, physicians and healthcare providers and their behaviours [36]. For example, a peak in media attention regarding group A streptococcal (GAS) disease and its testing in paediatric emergency departments was associated with an increase in the prescription of rapid tests for GAS despite no increase in number of children presenting symptoms that might warrant such testing [37]. In another example, wide media coverage resulted in striking changes in the use of hormone therapy by postmenopausal women [38]. A Cochrane systematic review highlighted the impact of the mass media on health services utilization, with a consistent effect after planned campaigns and unplanned coverage [39]. A recent study of statins use highlighted the potential effect of widely covered health stories in the media on real-world behaviour related to healthcare [40].

### Limitations

This study has some limitations. First, the sheer amount of social media (Facebook posts/tweets) where the chance of missing information is possible and may not all be captured by Altmetric. Second, the power may be limited to detect a relationship between the study design and online media attention. Third, our search strategy was simple, relying on only the term “cancer” in all fields, but was very large and unspecific. Fourth, the search was performed with MEDLINE only because it is the most frequently used database, and we did not aim to perform a comprehensive search. Fifth, date extraction was limited to one reviewer for 75% articles. However, we assessed the quality of data extracted because a second reviewer independently extracted the data for 25% articles and the reproducibility was very good, with kappa coefficient >0.9. Sixth, the Altmetric score, which was registered at a fixed point, may have influenced the results. However, a major part of this influence is corrected by adjustment on post-publication exposure periods even if cumulation of Altmetric score over time is probably no linear. Seventh, our search period focused on the first 6 months of 2014 because we wanted to have sufficient delay since the launch of Altmetric, in 2012, and we aimed to have a post-publication exposure period (i.e., period from last publication date [June 30, 2014] to the Altmetric search date [May 1, 2015]) of at least 10 months to ensure that the Altmetric score would be stabilized for most articles. Finally, the results should be interpreted with caution because the RoM value for press releases had wide confidence intervals.

Further research is needed to measure the impact of cancer research on individual components of media such as news and social media.

## Conclusions

There is a large variability in online media coverage of articles evaluating cancer treatments. Most important factors associated with high online media attention are presence of a press release and journal impact factor. There was no evidence that study design with high level of evidence and type of abstract conclusion were associated with high online media attention.

## Additional files

Additional file 1: Kappa coefficients for concordance in screening titles and abstracts of articles. This word file gives the individual estimates of Kappa coefficients for concordance between two researchers in screening title and abstracts to include articles in the study (11.5 Ko). (DOCX 22 Kb)

Additional file 2: Criteria to calculate the Altmetric score. This word file provides the information that how Altmetric score is calculated and weighted (14.5 Ko). (DOCX 29 Kb)

Additional file 3: Journals including the selected articles. This word file gives the detail of included journals, selected number of articles in each journal and description of some articles which received high Altmetric score in related journal (30.0 Ko). (DOCX 39 kb)
