# Supplementary material for: Factors associated with online media attention to research: a cohort study of articles evaluating cancer treatments
Source: Res Integr Peer Rev. 2017 Jul 1;2:9. doi: 10.1186/s41073-017-0033-z (PMC5803628; doi:10.1186/s41073-017-0033-z)
Supplement: Supplementary file 1 — Kappa coefficients for concordance in screening titles and abstracts of articles. This word file gives the individual estimates of Kappa coefficients for concordance between two researchers in screening title and abstracts to include articles in the study (11.5 Ko). (DOCX 22 Kb) [file 41073_2017_33_MOESM1_ESM.docx]

**Additional file 1: Kappa coefficients for concordance in screening titles and abstracts of articles (n = 200)**

| **Items** | **Kappa** | **95%CI** |
| --- | --- | --- |
| Study design | 0.95 | [0.91;0.98] |
| Cancer type by organ | 0.98 | [0.96;1.00] |
| Type of treatment | 0.98 | [0.96;1.00] |
| Sample size reported (Y/N) | 0.89 | [0.80;0.98] |
| Type of abstract conclusion | 0.94 | [0.90;0.98] |
| Funding source | 0.93 | [0.89;0.97] |
| Open access to the article (Y/N) | 0.94 | [0.90;0.99] |
