# Supplementary material for: Factors associated with online media attention to research: a cohort study of articles evaluating cancer treatments
Source: Res Integr Peer Rev. 2017 Jul 1;2:9. doi: 10.1186/s41073-017-0033-z (PMC5803628; doi:10.1186/s41073-017-0033-z)
Supplement: Supplementary file 2 — Criteria to calculate the Altmetric score. This word file provides the information that how Altmetric score is calculated and weighted (14.5 Ko). (DOCX 29 Kb) [file 41073_2017_33_MOESM2_ESM.docx]

**Additional file 2: Criteria to calculate Altmetric score**

It is a standardized score and is calculated based on three criteria: first is *Volume;* that how many people are mentioning that article. The score for an article rises as more people mention it and it counts only 1 mention from each person per source. Second is *Source;* each source contributes a different base amount to the final score, e.g. a newspaper article contributes more than a blog post which contributes more than a tweet. Third is the *Author* of each mention that who mentioned the published articles, at whether or not there is any bias towards a particular journal or publisher and at who the audience is; for example a doctor sharing a link with other doctors counts for far more than a journal account pushing the same link out automatically [^1^](#_ENREF_1). If Altmetric score is zero, it means, the article did not receive any public attention.

**The score is a weighted count** [**^2^**](#_ENREF_2)

The score is derived from an automated algorithm, and represents a weighted count of the amount of attention which have picked up for a research output. Why is it weighted? To reflect the relative reach of each type of source. It's easy to imagine that the average newspaper story is more likely to bring attention to the research output than the average tweet. This is reflected in the default weightings:

| News | 8 |
| --- | --- |
| Blogs | 5 |
| Twitter | 1 |
| Facebook | 0.25 |
| Sina Weibo | 1 |
| Wikipedia | 3 |
| Policy Documents (per source) | 3 |
| Q&A | 0.25 |
| F1000/Publons/Pubpeer | 1 |
| YouTube | 0.25 |
| Reddit/Pinterest | 0.25 |
| LinkedIn | 0.5 |

The Altmetric score always has to be a whole number. This means that mentions that contribute less than 1 to the score sometimes get rounded up to one. So, if we picked up one Facebook post for a paper, the score would increase by 1, but if we picked up 3 more Facebook posts for that same article, the score would still only increase by 1. 
(LinkedIn and Pinterest have deprecated as sources, as they started putting more of their content behind login pages, which made it more difficult for us to pick up mentions from them).

**References**

1. Altmetric http://wwwaltmetriccom/whatwedophp. . 2012

2. Altmetric: How is the Altmetric Score calculated? https://help.altmetric.com/support/solutions/articles/6000060969-how-is-the-altmetric-score-calculated-. 2015
