# Supplementary material for: Factors associated with online media attention to research: a cohort study of articles evaluating cancer treatments
Source: Res Integr Peer Rev. 2017 Jul 1;2:9. doi: 10.1186/s41073-017-0033-z (PMC5803628; doi:10.1186/s41073-017-0033-z)
Supplement: Supplementary file 3 — Journals including the selected articles. This word file gives the detail of included journals, selected number of articles in each journal and description of some articles which received high Altmetric score in related journal (30.0 Ko). (DOCX 39 kb) [file 41073_2017_33_MOESM3_ESM.docx]

| **Additional file 3: Journals including the selected articles** | | | | | | | | | **Description of some articles with high Altmetric score** | | | | | | | |
| --- | --- | --- | --- | --- | --- | --- | --- | --- | --- | --- | --- | --- | --- | --- | --- | --- |
| **S/No** | **Type of Journal** | **Full Journal Title** | **Total Cites** | **Journal Impact Factor** | **Eigenfactor Score** | **Total No of Articles** | **Included articles** | **Excluded articles** | **Altmetric Score on May 1, 2015** | **PMIDs** | **Study design** | **Cancer type** | **Type of treatment** | **Type of conclusion** | **Fudning source** | **Open access to the article** |
| 1 | Cancer | CA-A CANCER JOURNAL FOR CLINICIANS | 16130 | 162,50 | 0,0603 | 18 | 0 | 18 |  |  |  |  |  |  |  |  |
| 2 | Medical | NEW ENGLAND JOURNAL OF MEDICINE | 257469 | 54,42 | 0,65797 | 13 | 6 | 7 | 191 | 24597866 | RCT | Prostate | Surgery | In favour | Non-profit | Yes |
| 3 | Medical | LANCET | 176528 | 39,21 | 0,38061 | 24 | 9 | 15 | 370 | 24333009 | RCT | Breasr | Hormone therapy | In favour | Profit | Yes |
| 4 | Cancer | NATURE REVIEWS CANCER | 36052 | 37,91 | 0,11207 | 39 | 0 | 39 |  |  |  |  |  |  |  |  |
| 5 | Medical | JAMA-JOURNAL OF THE AMERICAN MEDICAL ASSOCIATION | 124822 | 30,39 | 0,25083 | 14 | 3 | 11 | 89 | 24715074 | SR/MA | Lungs | Chemotherapy | In favour | Non-profit | Yes |
| 6 | Medical | NATURE MEDICINE | 60002 | 28,05 | 0,16292 | 9 | 0 | 9 |  |  |  |  |  |  |  |  |
| 7 | Cancer | The Lancet, Oncology | 20565 | 24,73 | 0,09311 | 96 | 58 | 38 | 88 | 24332238 | RCT | Digestive system | Chemotherapy | In favour | Non-profit | Yes |
| 8 | Cancer | JOURNAL OF CLINICAL ONCOLOGY | 130991 | 17,96 | 0,37162 | 235 | 121 | 114 | 428 | 24470004 | RCT | Breast | Supportive care | In favour | Non-profit | Yes |
| 9 | Medical | BMJ-British Medical Journal | 85434 | 16,38 | 0,15994 | 20 | 7 | 13 | 286 | 24916719 | Observational study | Breast | Supportive care | In favour | Non-profit | Yes |
| 10 | Medical | ANNALS OF INTERNAL MEDICINE | 47309 | 16,10 | 0,10256 | 15 | 0 | 15 |  |  |  |  |  |  |  |  |
| 11 | Cancer | Nature Reviews Clinical Oncology | 3523 | 15,70 | 0,02285 | 42 | 0 | 42 |  |  |  |  |  |  |  |  |
| 12 | Medical | Annual Review of Medicine | 5560 | 15,48 | 0,0148 | 11 | 0 | 11 |  |  |  |  |  |  |  |  |
| 13 | Cancer | JNCI-Journal of the National Cancer Institute | 37903 | 15,16 | 0,07242 | 96 | 10 | 86 | 319 | 24563519 | Observational study | Prostate | Supportive care | Against | Profit | Yes |
| 14 | Medical | Science Translational Medicine | 9222 | 14,41 | 0,06994 | 22 | 1 | 21 | 131 | 24760190 | Observational study | Digestive system | Supportive care | In favour | Non-profit | Yes |
| 15 | Medical | PLOS MEDICINE | 16975 | 14,00 | 0,07366 | 5 | 0 | 5 |  |  |  |  |  |  |  |  |
| 16 | Medical | JOURNAL OF EXPERIMENTAL MEDICINE | 64191 | 13,91 | 0,13866 | 5 | 0 | 5 |  |  |  |  |  |  |  |  |
| 17 | Medical | JOURNAL OF CLINICAL INVESTIGATION | 96908 | 13,77 | 0,19342 | 55 | 0 | 55 |  |  |  |  |  |  |  |  |
| 18 | Medical | ARCHIVES OF INTERNAL MEDICINE | 39734 | 13,25 | 0,08546 | 0 | 0 | 0 |  |  |  |  |  |  |  |  |
| 19 | Cancer | CANCER RESEARCH | 142970 | 9,28 | 0,25797 | 358 | 3 | 355 | 15 | 24795429 | Observational study | Brain | Others | In favour | Non-profit | Yes |
| 20 | Cancer | CLINICAL CANCER RESEARCH | 68311 | 9,14 | 0,01322 | 269 | 42 | 227 | 66 | 24443618 | RCT | Breast | Others | In favour | Profit | Yes |
| 21 | Medical | BMC Medicine | 4052 | 7,28 | 0,01736 | 9 | 2 | 7 | 5 | 24479409 | RCT | Digestive system | Others | In favour | Non-profit | Yes |
| 22 | Cancer | ANNALS OF ONCOLOGY | 24363 | 6,58 | 0,07518 | 143 | 57 | 86 | 25 | 24504442 | Phase I/II, non-RCTs | Others | Chemotherapy | In favour | Non-profit | Yes |
| 23 | Cancer | CANCER TREATMENT REVIEWS | 4867 | 6,47 | 0,01412 | 103 | 16 | 87 | 8 | 24268442 | SR | Lungs | Supportive care | In favour | Not reported | No |
| 24 | Medical | Cochrane Database of Systematic Reviews | 39856 | 5,94 | 0,13531 | 14 | 12 | 2 | 19 | 24414552 | SR | Others | Supportive care | In favour | Non-profit | Yes |
| 25 | Cancer | Breast cancer research : BCR | 8270 | 5,88 | 0,02823 | 27 | 3 | 24 | 5 | 24745601 | SR/MA | Breast | Supportive care | In favour | Not reported | Yes |
| 26 | Medical | MAYO CLINIC PROCEEDINGS | 9716 | 5,81 | 0,01906 | 16 | 1 | 15 | 129 | 24958698 | Observational study | Others | Supportive care | In favour | Non-profit | Yes |
| 27 | Medical | CANADIAN MEDICAL ASSOCIATION JOURNAL | 12130 | 5,81 | 0,02479 | 0 | 0 | 0 |  |  |  |  |  |  |  |  |
| 28 | Cancer | Journal of thoracic oncology : official publication of the International Association for the Study of Lung Cancer | 8282 | 5,80 | 0,03949 | 113 | 37 | 76 | 74 | 24445595 | Observational study | Lungs | Others | In favour | Non-profit | Yes |
| 29 | Medical | JOURNAL OF INTERNAL MEDICINE | 8511 | 5,79 | 0,01698 | 4 | 0 | 4 |  |  |  |  |  |  |  |  |
| 30 | Medical | AMERICAN JOURNAL OF MEDICINE | 22679 | 5,30 | 0,02806 | 8 | 3 | 5 | 11 | 24384102 | SR | Others | Others | Neutral | Non-profit | No |
| 31 | Cancer | NEURO-ONCOLOGY | 4478 | 5,29 | 0,01733 | 59 | 9 | 50 | 5 | 24627236 | Observational study | Brain | Others | Neutral | Non-profit | Yes |
| 32 | Cancer | Cancer Prevention Research | 4033 | 5,27 | 0,02136 | 0 | 0 | 0 |  |  |  |  |  |  |  |  |
| 33 | Cancer | INTERNATIONAL JOURNAL OF CANCER | 46009 | 5,01 | 0,09896 | 586 | 73 | 513 | 226 | 24470442 | Observational study | Breast | Supportive care | In favour | Non-profit | Yes |
| 34 | Cancer | Journal of Hematology & Oncology | 1124 | 4,93 | 0,00451 | 40 | 7 | 33 | 4 | 24642247 | Phase I/II, non-RCTs | Blood | Chemotherapy | In favour | Profit | Yes |
| 35 | Cancer | ENDOCRINE-RELATED CANCER | 4977 | 4,91 | 0,01333 | 82 | 6 | 76 | 1 | 24174370 | Observational study | Breast | Supportive care | In favour | Non-profit | Yes |
| 36 | Cancer | CANCER | 62604 | 4,90 | 0,10396 | 319 | 79 | 240 | 214 | 24375332 | RCT | Breast | Hormone therapy | In favour | Non-profit | Yes |
| 37 | Cancer | RADIOTHERAPY AND ONCOLOGY | 12480 | 4,86 | 0,03161 | 48 | 15 | 33 | 7 | 24906626 | Observational study | Liver | Radiotherapy | In favour | Non-profit | No |
| 38 | Cancer | Gastric Cancer | 2089 | 4,83 | 0,00434 | 75 | 25 | 50 | 3 | 24122094 | Phase I/II, non-RCTs | Digestive system | Radiotherapy | In favour | Non-profit | Yes |
| 39 | Cancer | EUROPEAN JOURNAL OF CANCER | 23038 | 4,82 | 0,05583 | 241 | 58 | 183 | 42 | 24613622 | Meta-analysis | Breast | Supportive care | In favour | Non-profit | No |
| 40 | Cancer | BRITISH JOURNAL OF CANCER | 39150 | 4,82 | 0,07834 | 441 | 84 | 357 | 180 | 24675385 | Observational study | Others | Supportive care | In favour | Non-profit | Yes |
| 41 | Medical | ANNALS OF MEDICINE | 3825 | 4,73 | 0,0086 | 8 | 1 | 7 | 1 | 24491173 | systematic Reviw | Others | Supportive care | Neutral | None | No |
| 42 | Medical | ANNALS OF FAMILY MEDICINE | 2896 | 4,57 | 0,01042 | 2 | 0 | 2 |  |  |  |  |  |  |  |  |
| 43 | Cancer | CANCER EPIDEMIOLOGY BIOMARKERS & PREVENTION | 20408 | 4,32 | 0,0506 | 91 | 14 | 77 | 13 | 24526287 | Observational study | Others | Supportive care | In favour | Non-profit | Yes |
| 44 | Medical | AMERICAN JOURNAL OF PREVENTIVE MEDICINE | 14189 | 4,28 | 0,03661 | 25 | 0 | 25 |  |  |  |  |  |  |  |  |
| 45 | Cancer | BREAST CANCER RESEARCH AND TREATMENT | 16303 | 4,20 | 0,05379 | 170 | 22 | 148 | 17 | 24554388 | RCT | Breast | Supportive care | In favour | Non-profit | Yes |
| 46 | Medical | Journal of Translational Medicine | 3947 | 3,99 | 0,01574 | 57 | 8 | 49 | 3 | 24708624 | Phase I/II, non-RCTs | Digestive system | Others | In favour | Non-profit | Yes |
| 47 | Medical | JOURNAL OF GENERAL INTERNAL MEDICINE | 13142 | 3,42 | 0,03268 | 11 | 0 | 11 |  |  |  |  |  |  |  |  |
|  |  |  |  |  |  | **4038** | **792** | **3246** |  |  |  |  |  |  |  |  |
